# An Original Asteraceae Based Infused Drink Prevents Metabolic Syndrome in Fructose-Rat Model

**DOI:** 10.3390/antiox12020340

**Published:** 2023-01-31

**Authors:** Arezki Yanis Idres, Didier Tousch, Claudie Dhuyque-Mayer, Imane Hammad, Karen Lambert, Guillaume Cazals, Karine Portet, Karine Ferrare, Luc. P. R. Bidel, Patrick Poucheret

**Affiliations:** 1 UMR 95 Qualisud, University of Montpellier, CIRAD, SupAgro Montpellier, BP 14491, CEDEX 5, 34093 Montpellier, France; 2 PhyMedExp, INSERM U1046, CNRS UMR 9214, University of Montpellier, CEDEX 5, 34295 Montpellier, France; 3 Laboratoire de Mesure Physique, University of Montpellier, Place Eugène Bataillon, CEDEX 5, 34093 Montpellier, France; 4 INRA, UMR AGAP, CIRAD, SupAgro, 2 Place Pierre Viala, 34060 Montpellier, France

**Keywords:** metabolic syndrome, bitter Asteraceae, (*Taraxacum officinale* W.) leaves, *Arctium lappa* L. roots, fructose-rat model, chlorogenic acid, chicoric acid

## Abstract

Metabolic syndrome (METS) is a complex disorder that predisposes an affected person to an increased risk of diabetes and cardiovascular disease. Bitter Asteraceae plants contain several compounds active against METS that can be used as an alternative preventive therapy. Our previous work showed that a natural chicory extract (NCRAE) containing chicoric acid (CRA) and chlorogenic acid (CGA) in a molar ratio of 70/30 exhibited an antioxidant, insulin sensitization and anti-hyperglycemic effect. The present study was designed to evaluate the preventive effects of an NCRAE-like extract against METS in a complementary natural pharmacotherapeutic approach. An original Asteraceae infused drink containing the NCRAE CRA/CGA molecular ratio equivalent was prepared from dandelion (*Taraxacum officinale* L.) and burdock (*Arctium lappa* L.). The anti-METS effect of this drink was evaluated on the fructose-rat model for 8 weeks. Body weight, blood biochemistry, hepatic glucose-6-phosphatase, arterial blood pressure glucose and insulin tolerance were evaluated after 8 weeks. Our results show that daily oral intake of the Asteraceae infused drink led to a reduction of body weight gain, hyperglycemia, hypertriglyceridemia, insulin resistance and hypertension. Moreover, rat-by-rat analysis of the insulinemia measures revealed two types of responders. One sub-group of subjects demonstrated normal insulinemia and the other subgroup demonstrated hyperinsulinemia. This hyperinsulinemia, associated with the inhibition of the glucose-6-phosphatase activity in the liver tissue, may suggest an insulin release caused by CGA. The present study suggests that this original infusion of dandelion leaves and burdock roots may be used as an adjuvant therapy to prevent metabolic syndrome.

## 1. Introduction

Metabolic syndrome (METS) is a cluster of biochemical abnormalities that increases the risk of type 2 diabetes and/or cardiovascular diseases [1]. METS affects 20–30% of adult populations worldwide and reaches 50% of people over 60 years in the US [2]. METS leads to insulin resistance via abdominal obesity, inflammation and oxidative stress. Abdominal obesity is caused by excessive energy storage in adipose tissue, leading to a multitude of metabolic disorders, including increased blood levels of free fatty acids, pro-inflammatory adipokines and reactive oxygen species (ROS). This last disorder disrupts the insulin signaling cascades and initiates a state of insulin resistance which may leads to type 2 diabetes [3,4,5].

The main management strategies for METS are based on lifestyle therapy including dietary changes and regular physical activity [6]. Often pharmacological therapy must be prescribed in order to act on one target: metformin treatment for its insulin sensitizing effect, Orlistat^®^ as a gastric lipase inhibitor or beta-blockers and calcium channel blockers for hypertension [7]. Actually, no chemical compound is currently known to act on the syndrome in its globality.

In this regard, plants with their great diversity of compounds, often pharmacologically active, have a good potential to be an alternative approach in METS care. A lot of substances have anti-inflammatory, anti-oxidative and anti-insulin resistance effects. Together, these three properties could break the pernicious circle of oxidative stress/inflammation and insulin-resistance which sets up slowly with self-amplification [8].

Many bitter Asteraceae that are commonly consumed are particularly interesting because of their potential to prevent METS. These plants are rich in sesquiterpene lactones and hydroxycinnamic acid esters [8]. Many hydroxycinnamic acid esters such as *trans-*caffeic acid (CA), *trans-*chlorogenic acid (CGA) and *L-*chicoric acid (CRA) are known for their pleiotropic biological activities both antioxidant [9,10], anti-inflammatory [11,12] and insulin sensitizing [13,14,15].

In a previous work by our research group, a root extract from *Cichorium intybus* (NCRAE) rich in chicoric acid (CRA) and chlorogenic acid (CGA) at a 70/30 molar ratio showed its capacity to induce, on in vitro model, an insulin sensitizing effect and a H_2_O_2_ protective effect in pretreatment in L6 muscular cells [15,16]. Additionally, in vivo experiments on streptozotocin diabetic rats have shown that NCRAE intraperitoneal administrations induced a clear anti-diabetic activity principally through an insulin-sensitizing effect. Moreover, the direct implication of CRA and CGA in the NCRAE anti-diabetic properties was demonstrated using a mixture of molecular standards of CRA and CGA (in the equivalent molar ratio of 70/30) which showed the same positive effects on streptozotocin diabetic rats [17].

The present study was designed to evaluate the preventive effects of a chronic oral administration NCRAE-like extract against insulin resistance and METS in the framework of a complementary natural pharmacotherapeutic approach.

In this context, an Asteraceae infused drink prepared by infusing dried dandelion *(Taraxacum officinale* F. H. Wigg) leaves and dried burdock (*Arctium lappa* L.) roots in water with a CRA/CGA molar ratio of 70/30, similar to the NCRAE ratio, was prepared and administered daily for 8 weeks to fructose-fed rats; a well-known animal model of METS. A comparison of the blood biochemistry, insulin and glucose tolerance parameters between the control cohort receiving a normal diet and the fructose cohorts, supplemented or not with the infusion, allowed us to evaluate the infusion effect on METS. This article describes for the first time the effects of the CRA/CGA ratio after oral administration, thereby taking into account the impact of the intestinal barrier on CRA and CGA absorption.

An analysis rat-by-rat of the physiological parameters allowed us to compare the global animal responses to the Asteraceae infused drink diet. Some animals had a close NCRAE-like response whereas the others showed a predominant CGA effect. To confirm this fact, we evaluated the hepatic glucose-6 phosphatase (G6Pase) activities in liver tissues collected from the animals, since CGA is a well-known inhibitor of this enzyme [17].

## 2. Materials and Methods

### 2.1. Materials

Dried fragments of burdock root (*Arctium lappa* L.) and dandelion leaves (*Taraxacum officinale* F. H. Wigg) were purchased from a certified company: Nature valley (Maine-et-Loire; France).

Fructose, glucose, chlorogenic acid (*trans-5-O-*caffeoylquinic acid) and *L-*chicoric acid (*2,3-O-*di*-O-*caffeoyl-*L-*tartaric acid) standards were from Sigma-Aldrich (Saint-Louis, MO, USA), while Metformin, the glucometer Bayer Health Ascensia Contour (Ile de France, Paris), glucose indicator strips and insulin were obtained in a local pharmacy (Pharmacie des vignes, Montpellier, France).

Male Wistar genotype rats of five weeks old (≈200g) were obtained from Janviers Labs (Le Genest-Saint-Isle, France).

### 2.2. Methods

#### 2.2.1. Infused Drink Preparation

A preliminary step allowed us to optimize the amount of plants to mix in order to obtain the proper CGA/CRA ratio equivalent to NCRAE [17]. The dried leaves and root fragments were immersed in equal parts (*w/w*) in boiling water for 3 min, followed by a 30 min rest without stirring. The resulting infused drink was then filtered and stored at 4 °C in the dark.

#### 2.2.2. Chemical Analysis by HPCL and LC-MS

The infused drink was analyzed by HPLC using an Agilent 1200 series (Agilent, Santa Clara, CA, USA) equipped with a Nova-pak HR C18 250 mm × 4.6 mm × 5 µm column (Waters Corps, Milford, CT, USA) and coupled with a DAD detector. The column thermostat was set to 25 °C, and the samples were injected automatically with an auto-sampler from the same manufacturer and with the volume programmed at 20 µL. The gradient was set with two phases. Phase A was composed of acetic acid 1%. Phase B was composed of acetonitrile. The program was as follows: time 0 was 100% A; from 0 to 15 min 40% A and 60% B; from 15 to 18 min 20% A and 80% B; from 18 to 25 min 100% A. The amount of CRA and CGA were quantified using a chromatographic peak area at 326 nm.

The chemical composition of the infused drink was characterized by LC-MS using a Synapt G2-S high-definition mass spectrometry system (Waters Corp., Milford, MA, USA) equipped with an electrospray ionization source, to characterize the elemental composition of parent and fragment ions according to the procedure previously described [17].

To characterize the elemental composition of the parent and fragment ions according to the procedure previously described [17], the chemical composition of the infused drink was characterized by LC-MS using a Synapt G2-S high-definition mass spectrometry system (Waters Corp., Milford, MA, USA) equipped with an electrospray ionization source. Chromatographic separation was carried out at a flow rate of 0.4 mL min^−1^ on the Acquity H-Class ultrahigh performance liquid chromatography (UPLC) system (Waters Corp., Milford, MA, USA), equipped with a Kinetex C18 100 A column (100 × 2.1 mm, 2.6 µm beads) from Phenomenex (Le Pecq, France). The mobile phase consisted of permuted water (solvent A) and acetonitrile (solvent B), both phases acidified by 0.1% (*v/v*) formic acid. Mass spectra were acquired in the negative ionization mode with a capillary voltage of 3 kV. Tandem mass spectra were acquired in the Fast-DDA (Data Directed Analysis) mode so that the two most abundant ions in the full scan MS would trigger tandem mass spectrometry (MS^2^). The TOF mass analyzer was calibrated using phosphoric acid in 1/1 (*v/v*) acetonitrile/H_2_O from 50 to 1500 *m/z* to obtain mass accuracy within 3 ppm. The Synapt parameters were optimized using the CGA standard as follows: the sample cone was set at 20 V, the source temperature and desolvation temperature were set at 120 and 600 °C, respectively. Each sample was processed with MassLynx (V4.1) software.

Peaks obtained are listed in Table 1 in the order of elution, with their parent ion and their fragments. Their molecular formulas were assessed using MassLynx 4.2 software, and its monoisotopic mass-to-charge ratio were compared to the theoretical one. The three main hydroxycinnamic acid esters (CA, CRA, CGA) were unequivocally identified on the basis of their concordance with retention times, UV absorbance spectra, the mass-to-charge ratio of the parent ions, and MS2 fragment proportions to pure authentic standards. Other peaks were tentatively assigned based on bibliographic data [17]. The compound abundance of each hydroxycinnamic acid ester was compared among samples using the absorbance peak area.

#### 2.2.3. In Vivo Experiment

##### Experimental Procedure

The animals (Wistar rats) were kept in a temperature-controlled room (20–22 °C) with a 12-h darkness-light cycle. They were accommodated three per cage where they were fed with a standard A04 SAFE-Augy-France diet and water ad libitum. The experiments were carried out in accordance with the rules of ethics and animal welfare. The experimental protocol has been approved by the Languedoc-Roussillon ethics committee (Authorization *n* APAFIS#2386-20200101409859 v3).

After an acclimatization period of one week, the animals were randomly divided into a control cohort (Control) (*n =* 6) receiving a conventional diet and into three fructose groups receiving 10% (*w/v*) fructose/water to induce an experimental METS [18]. The fructose groups differed according to the treatment: the untreated fructose group (Fructose-NT) (*n =* 6), the fructose group treated with the Asteraceae infused-drink (500 mg·kg^−1^·daily^−1^) (*n* = 8) (Fructose-AT) and the fructose group treated with metformin (100 mg·kg^−1^·daily^−1^) (*n* = 6) (Fructose-MT) as drug control.

##### Weight Evolution

After random affectation of rat to the various groups, the animals’ weight was measured each week throughout the experiment.

##### Glycemia, Insulinemia and Triglyceride Levels

The plasma glucose level (mg·dL^−1^) was measured using the BIOLABO colorimetric glucose assay kit, ref. 80009 (Maizy, France). The plasma insulin level (ng·mL^−1^) was determined using the rodent insulin ELISA kit purchased from ALPCO (Eurobio-Ingen, les Ullis, France). The plasma triglycerides (TG) level was analyzed using the BIOLABO kit ref. 90406-90416- (Maizy, Aisne, France).

##### Oral Glucose Tolerance Test (OGTT)

The OGTT was carried out after 8 weeks. After a 5 h fasting period, a glucose solution was administered orally (3 g·kg^−1^). Blood was taken from the caudal vein of the rats at different times (0, 15, 30, 60, 120 and 180 min). Glycemia was determined using a glucometer (Accu-Chek Guide, Roche, Switzerland) which was calibrated before each use with a control solution (Accu-Chek Ref 07748906).

##### Insulin Tolerance Test (ITT)

The ITT was carried out at 8 weeks. After a 5 h fasting period, insulin (0.5 IU·kg^−1^) was administered by intra-peritoneal injection and blood was taken from the caudal vein of the rats at different times (0, 15, 30, 60 and 120 min). The blood glucose level was determined using the glucometer.

##### Blood Pressure Measurement

Rats were taken out from the animal room and brought into a quiet separate laboratory. The method used was the indirect measurement of systolic blood pressure (tail cuff) without external preheating. The equipment (Harvard Apparatus, Les Ullis, France) included animal holders, a manual scanner, a pulse amplifier, a tail cuff, and a central unit for digital recording. Ambient temperature was stabilized at 30 °C. Animals were conditioned to this assay procedure to obtain readings. Systolic blood pressure was measured in conscious animals and the mean of five readings was considered as systolic blood pressure measurement.

#### 2.2.4. In Vitro Experiment

##### Microsome Purification

At the end of the 8-week experiment, the rats were euthanized, and the liver was taken and stored at −80 °C. The liver (1 g) was delicately ground in a potting vessel containing a lysis buffer (250 mM sucrose; 50 ug mL DNase I; 1 mM MgCl_2_; 4 mM HEPES; Protease inhibitor 4 µM (Pepstatin, Aprotinin, PMSF)). The ground livers were centrifuged at 10,000× *g* for 10 min at 4 °C. The recovered supernatant was lysed in nitrogen (55 bar, 15 min with agitation) and then placed on a sucrose gradient (0.8 to 2.0 M) and centrifuged at 25,000× *g* for 16 h at 4 °C. The different fractions were recovered independently, and the proteins content were assayed with a Bradford reagent. The fractions with the highest G6pase activity were used for the experiment.

##### Glucose-6-Phosphatase Activity

The glucose-6-phosphatase activities were obtained using the protocol previously described [16,19] with some modifications. Briefly, 2 µg of microsomal suspension were incubated for 30 min in 20 mM glucose 6 phosphate buffer (20 mM glucose-6-phosphate; 250 mM sucrose; 500 mM HEPES). The reaction was stopped, and Pi was determined by the addition of a malachite green reagent (1 part 4.2% ammonium molybdate in 3 parts 0.2% malachite green; 5 mol/L H_2_SO_4_ and 0.1% Tween 20). After 20 min incubation at room temperature, the optical density was measured at 660 nm. A calibration curve with different NaH_2_PO_4_ concentration was established. Results were expressed as *n* moles of inorganic phosphate produced in 1 h for 2 µg of protein measured. Chlorogenic acid (50 µg mL^−1^) was used as a positive control to verify membrane integrity.

##### Statistical Analysis

Data are expressed as means ± S.E.M. and were analyzed using the XLSTAT Premium software by Addinsoft. One-way ANOVA was performed for data followed by Fisher’s LSD post hoc test for multiple comparisons. A value of *p* < 0.05 was considered as statistically significant.

## 3. Results

### 3.1. Chemical Composition Analysis of the Asteraceae Infused-Drink by LC-MS

The chemical analysis of this infused-drink (Table 1) revealed the presence of three caffeoyl-tartaric acid esters (P2, P8 and P9), originating from the *Taraxacum* fraction. Peak 8 co-eluted with the *L-*chicoric acid standard and had both a similar absorbance spectrum and a similar fragmentation pattern. Peak 9 had at least the same fragmentation pattern and may correspond to one of its diastereoisomers, already described in literature [20,21]. The infused drink contained a large amount of chlorogenic acid (*trans-5-O*-caffeoylquinic acid) and 10 additional caffeoylquinic acid esters in a lower proportion. Based on their 326-nm-absorbance integrals, we deduced that the molar ratio of CGA to the total of CRA isomers was approximately equivalent to (30/70).

Since flavonoids and sesquiterpenes are known to have potential anti-hyperglycemic bioactivities, we tried to identify them in our infused drink. We observed that immersing parts of plants in boiling water for 3 min followed by a rest of 30 min without stirring was not long enough to extract flavonoids of *Taraxacum officinale* (quercetin diglycosides and trigycosides, luteolin diglycosides, chrysoeriol diglycosides), listed in [21,22], of *Arctium lappa* (quercetin rhamnoside, quercetin*-3-O-*glucuronide and quercetin*-3-O-*vicianoside), listed in [23,24]. By contrast, these flavonoids were present in methanolic extracts of the same powdered sample (data not shown). We also verified that the sesquiterpenes of *Taraxacum officinale* leaves described by [25] i.e., taraxacolide esters and taraxinic acid derivatives, were not extracted. Similarly, sesquiterpenes of *Arctium lappa* previously isolated from shoots [24], were not found in our extract. The extraction medium was not enough apolar for extracting flavonoids and sesquiterpenes of the two species. In conclusion, this infused drink was mainly composed of L-chicoric acid, *1,5-O-*dicaffeoyl-*4-O-*malonylquinic acid and chlorogenic acid, associated with minor caffeoylquinic acid isomers.

**Table 1:** UV spectra and characteristic ions of phenolic compounds found in the infusion mixture of dandelion leaves (*Taraxacum officinale* WEB. Ex WIGG) and burdock taproot (*Arctium lappa* L.).

Peak	RT (min)	Abs 326 nm%	UV λmax/λmin (nm)	Tentative identification	Parention	Monoisotopic ratio *m/z*		Masse deviation	Fragmentation data m/Z (% base peak)	References
						Theo *m/z*	Precision *m/z*			
P1	4.20	0.6	298sh, 324	*1-O-*caffeoylquinic acid	C_16_H_11_O_9_^−^	353,0873	353,0871	0.6	191(100),179(67), 93(64)	[22,26,27]
P2	5.25	2.8	241, 310sh, 327	*2-O-*caffeoyl-*L-*tartaric acid (caftaric acid)	C_13_H_11_O_9_^−^	311,0403	311,0402	0.3	149(100), 179(40), 135(5)	[21,22]
P3	8.90	4.7	298sh, 325	*3-O-*caffeoylquinic acid	C_16_H_17_O_9_^−^	353,0873	353,0875	−0.6	179(75), 161(10), 135(19), 85(25)	[22,27]
P4	19.10	17.7	252, 304sh, 324	*5-O-*caffeoylquinic acid (chlorogenic acid)	C_16_H_17_O_9_^−^	353,0873	353,0872	0.3	191(100), 85(12)	[20,22,27]
P5	26.44	0.8	240, 306sh, 324	*4-O-*caffeoylquinic acid	C_16_H_17_O_9_^−^	353,0873	353,0875	−0.6	173(100), 191(34), 179(75), 135(26)	[22,27]
P6	32.20	0.1	240, 302sh, 324	*1,4-di-O-*caffeoylquinic acid	C_25_H_23_O_12_^−^	515,119	515,1189	0.2	353(100), 299(37), 255(28), 203(61), 179(3), 173(19)	[20,22,27]
P7	52.67	1.1	243, 303sh, 327	*3,5-di-O-*caffeoylquinic acid	C_25_H_23_O_12_^−^	515,119	515,1189	0.2	353(100), 191(12), 179(9), 85(10)	[20,22,27]
P8	54.20	31.9	242, 305sh, 328	*(2S, 3S)-di-O-*caffeoyltartaric acid (*L-*chicoric acid)	C_22_H_17_O_12_^−^	473,072	473,0721	−0.2	311(100), 293(45), 149(25), 179(23), 312(15), 294(6), 219(8), 341(4), 135(4)	[21,22]
P9	55.05	23.2	242, 305sh, 328	*(2R, 3R)-di-O-*caffeoyltartaric acid (chicoric acid isomer)	C_22_H_17_O_12_^−^	473,072	473,0719	0.2	311(100), 293(46), 149(26), 179(21), 312(14), 294(8), 219(7), 341(5), 135(3)	[21,22]
P10	55.45	6.4	217, 307sh, 327	*1,5-di-O-*caffeoyl*-4-O-*maloylquinic acid	C_29_H_27_O_16_^−^	631,1299	631,1298	0.2	515(100), 469(91), 353(100), 191(95), 179(15), 161(11), 135(8), 111(10)	[20,26]
P11	55.81	2.1	216, 306sh, 328	*1,5-di-*O-caffeoyl*-3-O-*succinoylquinic acid	C_29_H_27_O_15_^−^	615,135	615,1348	0.3	515(15), 453(25), 353(100), 335(5), 191(25), 127(14), 93(12), 85(10)	[21,26,27]
P12	56.20	1.7	214, 310sh, 326	*1,5-di-O-*caffeoylquinic acid	C_25_H_23_O_12_^−^	515,119	515,1191	−0.2	353(100), 191(10), 173(25), 179(10)	[26,27]

Peaks are listed in the order of elution, with their names, molecular formula before ionization (RT), and retention times; Mol. form.: precursor ions, and fragmentation data of hydroxycinnamoylquinic acids. Theo. mass: theoretical monoisotopic mass of precursor ion [M − H]^−^. Δppm: mass tolerance expressed in parts per million.

### 3.2. In Vivo Experiment

#### 3.2.1. Animal Weight Evolution

**Figure 1:**
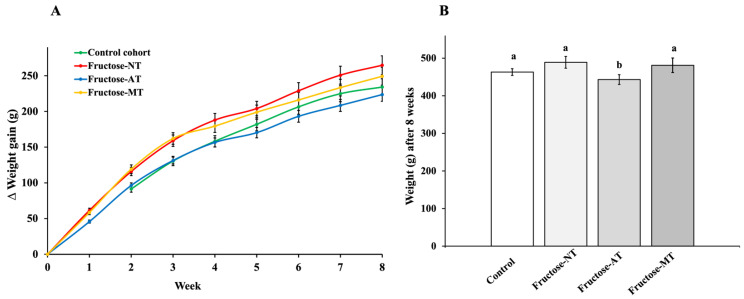
Weight of animals during the experimentation. (**A**) Time course showing the weight gain of animals during the 8 weeks. (**B**) Bar graph showing the weight of animals at the end of the experiment. Different letters show a significant difference at (*p* < 0.05).

Weight evolution over the 8 weeks of the experiment was slightly elevated (not statistically significant) for fructose-NT rats versus control rats. Metformin treatment did not modify the evolution (Figure 1A). In contrast, the chronic Asteraceae infused drink induced a significant decrease in body weight starting at the fourth week. This effect was maintained at the eighth week (*p* < 0.05).

At the eighth week (Figure 1B), the Fructose-NT rats had a higher body weight (489 ± 15.2 g) than the control rats (463 ± 8.8 g). The daily Asteraceae infused drink induced a significant decrease (*p* < 0.05) in body weight of the Fructose-AT (443 ± 12.9 g) rats when compared to Fructose-NT. Metformin treatment in fructose rats led to a nonsignificant decrease in weight (481 ± 19.2 g) compared to the Fructose-NT rats.

#### 3.2.2. Biological Parameters

**Figure 2:**
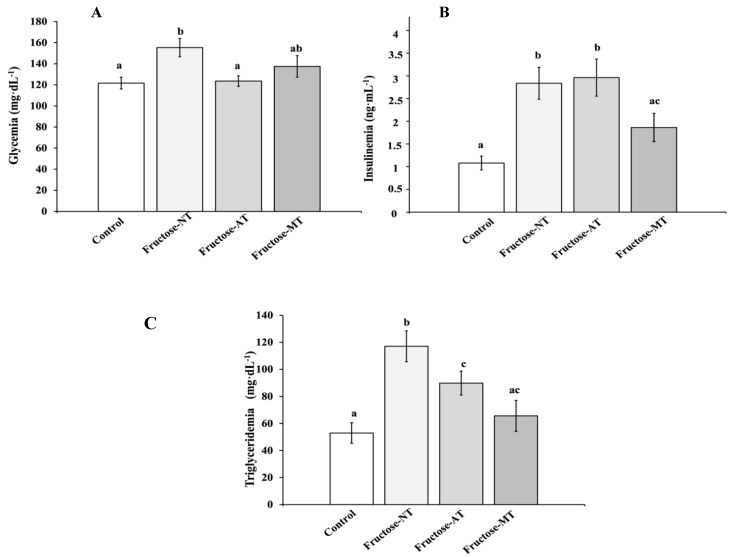
Biochemical parameters evaluated at the end of the experiment (week 8). (**A**) Blood glucose (**B**) Insulinemia (**C**) Triglycerides levels. Different letters show significant differences at (*p* < 0.05).

Glycemia (Figure 2A): At the eighth week, the fructose beverage induced a clear hyperglycemia (155 mg·dL^−1^ ± 8.6) compared to control rats (121 ± 5.5 mg·dL^−1^) (*p* < 0.05). The daily administration of the Asteraceae infused drink or metformin reduced significantly (*p* < 0.05) the fructose induced hyperglycemia to be significant in Fructose-AT (123 ± 4.9 mg·dL^−1^) and in Fructose-MT (134 ± 10 mg·dL^−1^). Glycemia correction was clearer in the Fructose-AT rats group.

Insulinemia (Figure 2B): At the eighth week, fructose administration significantly increased (*p* < 0.05) the plasma insulin level at 2.8 ± 0.35 ng·mL^−1^ in Fructose-NT rats versus 1.08 ± 0.15 ng·mL^−1^ for control rats. Metformin treatment significantly decreased fructose induced hyperinsulinemia to 1.86 ± 0.31 ng·mL^−1^ (*p* < 0.05). Surprisingly, the fructose hyperinsulinemia was not reduced by the Asteraceae infused drink (2.9 ± 0.40 ng·mL^−1^).

Triglyceridemia (Figure 2C): After 8 weeks, the fructose beverage provoked a hyper-triglyceridemia (*p* < 0.05) in fructose-NT rats (117 ± 12.5 mg·dL^−1^) versus (52 ± 7.6 mg·dL^−1^) for control rats. The hyper-triglyceridemia was reversed in fructose-MT rats (67 ± 12.8 mg·dL^1^). With the Asteraceae infused drink, a significant decrease was also observed (87 ± 8.1 mg·dL^−1^) (*p* < 0.05) but less than with the metformin treatment.

#### 3.2.3. Oral Glucose Tolerance Test (OGTT)

**Figure 3:**
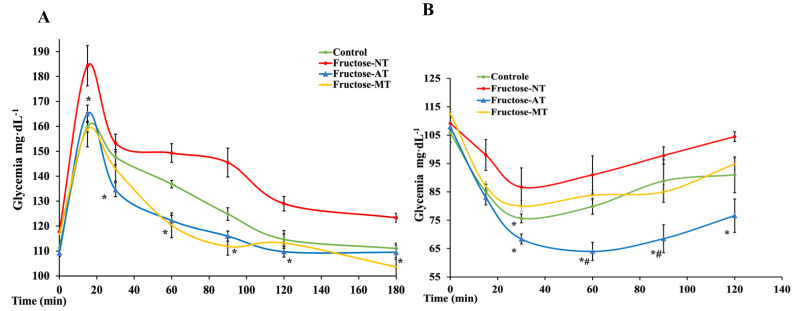
Tests of glucose and insulin tolerance at the end of experiment. (**A**) Oral glucose tolerance. (**B**) Intraperitoneal insulin tolerance. *: significant differences compared to Fructose-NT. #: significant differences compared to control (*p* < 0.05).

At the eighth week of the experiment, an oral glucose intake provoked in rats a hyperglycemic peak after 15 min for all groups. This glucose peak level is significantly (*p* < 0.05) higher in fructose rats (184 ± 8.0 mg·dL^−1^) compared to the control (159 ± 2.1 mg·dL^−1^). Chronic metformin administration or Asteraceae infused drink intake in fructose rats significantly (*p* < 0.05) reduced the hyperglycemic peak to values close to normal (158 ± 6.9 mg·dL^−1^ and 165 ± 3.7 mg·dL^−1^, respectively) (Figure 3A). The return to basal glycemic values was slower for fructose rats when compared to control rats. Treatment with Asteraceae and metformin allowed a faster return to the basal blood glucose value.

#### 3.2.4. Insulin Tolerance Test (ITT)

At the eighth week of the experiment, an intraperitoneal injection of insulin induced a clear decrease in glycemia in control rats (Figure 3B). Blood glucose level decreased from 105 ± 3.3 mg·dL^−1^ to 75 ± 1.5 mg·dL^−1^ at 30 min and then gradually rose back to basal value. In fructose treated rats (Fructose-NT), an insulin load induced a smaller reduction in blood glucose reaching 86.6 ± 6.7 mg·dL^−1^ at 30 min, a rapid return to normal blood glucose was observed. In metformin treated rats (Fructose-MT), the drop of glycemia after insulin load was similar to the values observed with the control rats (80 ± 1.9 mg·dL^−1^). As for the daily treatment by the Asteraceae infused –drink, ITT was characterized by a drastic fall in glycemia, reaching 68 ± 4.8 mg·dL^−1^ at 30 min. This hypoglycemia lasted after the 120 min endpoint.

**Figure 4:**
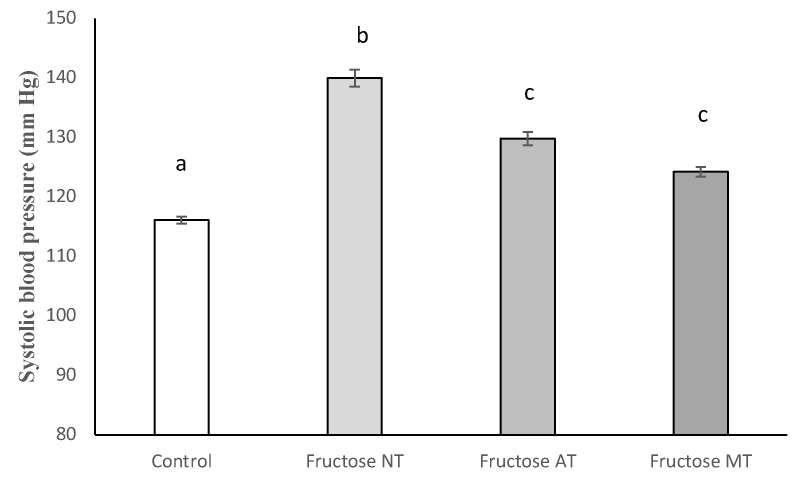
Systolic arterial blood pressure at the end of the experiment (week 8). Different letters show significant differences at (*p* < 0.05).

Arterial systolic blood pressure (SBP) measured at the eighth week of the experiment (Figure 4) showed, as expected, a statistically significant elevation for the Fructose-NT group when compared with the control group of animals. Fructose-MT rats, treated with metformin, demonstrated a significant decrease of SBP when compared to the Fructose-NT group. This observation validated the assay since metformin was used in an internal standard in this assay. Similarly, the Fructose-AT group’s SBP was significantly decreased to a statistically similar level than the Fructose-MT group. Neither group reached the full correction of SBP as they remained slightly above the Control group.

#### 3.2.5. Specific Rat-by-Rat Analysis of Results Obtained with Asteraceae Infused Drink

**Figure 5:**
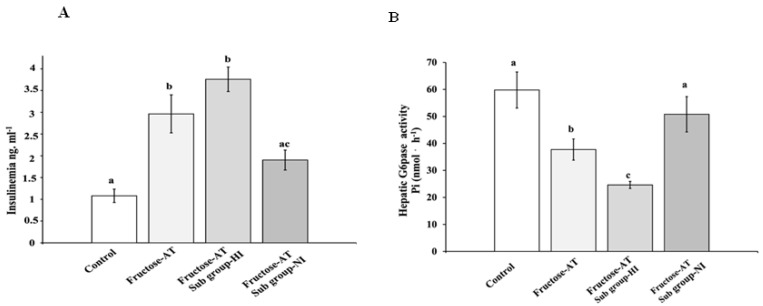
Specific analysis of the two subgroups of the Asteraceae infused drink. (**A**) Insulinemia; (**B**) Hepatic glucose-6-phosphatase activities. Subgroup-HI (high insulinemia) (*n* = 5); subgroup-NI (normal insulinemia) (*n* = 3). Different letters (a, b and c) indicate significantly differences from each (*p* < 0.05).

**Figure 6:**
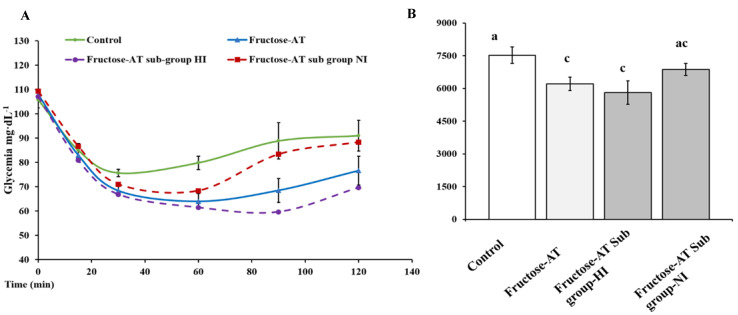
Specific analysis of the two subgroups of Asteraceae infused drink. (**A**) Intraperitoneal insulin tolerance test (**B**) Subgroup-HI (high insulinemia) (*n* = 5); subgroup-NI (normal insulinemia) (*n* = 3). Different letters (a and c) indicate significantly differences from each (*p* < 0.05).

The analysis of insulinemia of fructose rats treated by the Asteraceae infused-drink revealed the identification of two subgroups: the first with a clear hyperinsulinemia, subgroup-HI (*n* = 5), the second subgroup with normal insulinemia, subgroup-NI (*n* = 3) (Figure 5A). Consequently, ITTs were different: hypoglycemia in HI rats was more important and persistent than in NI rats (Figure 6). The final number of animals per subgroups was lower than the initial complete Fructose-AT cohort, which was *n* = 8. So, it will be necessary to confirm this result with a more important animal’s cohort in our future investigations. Nonetheless, the differential insulinemia between the two subgroups is sufficiently significant to consider this tendency. Glucose-6-phosphatase activity of hepatic microsomal fraction was clearly reduced (*p* < 0.05) in all rats treated by the Asteraceae infused drink. However, in HI rats this activity was significantly lower than the one observed in NI rats (Figure 5B).

## 4. Discussion

The present study demonstrated that a supplementation with plant extract from dandelion leaves and burdock roots rich in hydroxycinnamic acid esters, particularly in CGA and CRA in a 70/30 molar ratio, could prevent METS induced by fructose in rats. The infusion of the dried parts of plants allowed us to obtain essentially the hydroxycinnamic acid esters content without the sesquiterpene lactones. After eight weeks of the fructose diet, rats developed a cluster of METS abnormalities including hyperglycemia, hyperinsulinemia, hypertriglyceridemia, insulin resistance demonstrated by OGTT and ITT, as well as hypertension. Our results are in agreement with previous results published by several authors that used the fructose diet induced METS [11,28,29]. Metformin was used as a positive control to prevent hyperglycemia, hyperinsulinemia, and hypertension through its insulin-sensitizing effect. We also observed a triglyceridemia modulation as previously shown [30,31]. A slight (not statistically significant) body weight gain was recorded. The daily Asteraceae infused drink prevented most disorders induced by fructose, including hypertension, but with the exception of hyperinsulinemia. Moreover, a clear decrease in body weight gain was observed which is of interest in the management of obesity associated to METS. This result could be explained by the fact that dandelion is known for its ability to reduce adipogenesis and lipid accumulation [32] as well as, additionally, its diuretic virtue which may also contribute to limit weight gain [14,33,34,35]. The hyperinsulinemia observed in animals without insulin resistance correlated to a strong ITT response suggesting a stimulation of insulin release by the Asteraceae infusion-drink. We have previously shown that NCRAE (CRA/CGA) is able to increase insulin secretion and CGA alone is described as a marked insulin secretion stimulator [15,36]. This last result suggested the need for a rat-by-rat analysis. This more specific analysis allowed us to distinguish two subgroups of responders in animals supplemented with the Asteraceae infused drink. Although the numbers of animals by cohort are low due to the group splitting after completion of the experiment, the marked trend allowed us to distinguish two subgroups: the first is characterized by hyperinsulinemia and strong ITT (*n* = 5) in contrast with the second subgroup demonstrating normal insulinemia and normal ITT (*n* = 3). In accordance with the literature, regarding the first subgroup-HI, we can consider that the results recorded may be based on CGA effects. In contrast, we revealed a clear decrease of hyper-insulinemia for the second subgroup-NI suggesting a more complex response involving potential synergic actions of the two caffeic acid derivatives leading to an insulin sensitization effect and a slight insulin secretion stimulation in accordance with the NCRAE effect [17]. To test the hypothesis of a predominant CGA effect in the subgroup-HI, we investigated the hepatic microsomal G6Pase activities as a specific CGA target. CGA has been recognized as a hepatic G6Pase inhibitor [37,38,39]. We observed a normal hepatic G6Pase activity exactly similar to an NCRAE-like effect previously reported [16,17] in the subgroup-NI (normal insulinemia) whereas in subgroup-HI (hyperinsulinemia), a significant decrease in hepatic G6Pase activity was observed. The existence of these two subgroups might be explained by an inter-individual disparity of CGA and CRA bioavailability. In fact, if CGA bioavailability is largely discussed [40,41,42,43], there is little data available on CRA bioavailability. Nonetheless, the hyper-insulinemia CGA-like effect can be considered as beneficial due to its hypoglycemic action.

In addition to the overall data obtained in the present assay, it should be mentioned that the original infused drink containing NCRAEs and its CRA/CGA molecular ratio are providing high protection against oxidative stress. This potential was demonstrated to be associated with antidiabetic effects [16,17] and thereby contribute to the beneficial effects recorded in the present study on METS.

## 5. Conclusions

In conclusion our original Asteraceae infused drink processed from dandelion leaves and burdock roots extracts did demonstrate beneficial properties to prevent METS. The molar ratio 70/30 was confirmed to be efficient in inducing the observed biological effects *in vivo*. A combined influence on insulin secretion and a potential reduction of adiposity may have contributed to the overall bioactivity. As well, the extended hypoglycemia recorded during the ITT challenge suggested an elevated insulin sensitizing effect. This study opens the way to other perspectives (1) an in depth molecular and metabolomic exploration of synergistic action of the CRA-CGA couple on insulin secretion, insulin sensitivity and antioxidant signaling pathways and (2) by a clinical trial on METS patients with obesity and elevated risk of cardiovascular disease.

Finally, this experiment also highlights the complexity of the response when a mix of compounds are used. These last effects could be correlated to the bioavaibility of each compound in the infusion. Therefore, it will be important, in our future studies, to investigate the complex interplays between compounds bioavailability and their combined bioactivities in natural plant extracts.

## Data Availability

Data is contained within the article.
